# System infection prevention in hospital networks in the United States—an SHEA research network inquiry into operational characteristics and current challenges

**DOI:** 10.1017/ice.2024.170

**Published:** 2025-01

**Authors:** Michael P. Stevens, Nkechi Emetuche, Catherine Passaretti, Graham Snyder, Rachael Snyders, Michael B. Edmond, Jonas Marschall

**Affiliations:** 1 West Virginia University School of Medicine, Morgantown, WV, USA; 2 Society for Healthcare Epidemiology of America, Arlington, VA, USA; 3 Atrium Health, Charlotte, NC, USA; 4 University of Pittsburgh, Pittsburgh, PA, USA; 5 BJC Healthcare, St. Louis, MO, USA; 6 West Virginia University School of Medicine, Morgantown, WV, USA; 7 Washington University School of Medicine, St. Louis, MO, USA; 8 BJC Healthcare, St. Louis, MO, USA

## Abstract

A 54-question survey about System Healthcare Infection Prevention Programs (SHIPPs) was sent out to SHEA Research Network participants in August 2023. Thirty-eight United States-based institutions responded (38/93, 41%), of which 23 have SHIPPs. We found heterogeneity in the structure, staffing, and resources for system infection prevention (IP) programs.

## Introduction

Healthcare consolidation has led to integrating IP into SHIPPs. Very little has been published about SHIPPs.

An informal survey of seven US-based healthcare epidemiologists published in 2022 revealed a number of factors facilitating the success of SHIPPs. These included adequate staffing, a single, integrated medical record system, data automation, and the ability for system personnel to interact with one another meaningfully. Barriers to optimally functioning SHIPPs identified included potential cohesion issues, heterogeneity in electronic medical record platforms, and rapidly expanding geographically widespread health systems.^1^

A major challenge in system approaches to IP includes limited access to infectious diseases trained physician healthcare epidemiologists to help lead these programs. Potentiating this problem is the fact that no formal certification in healthcare epidemiology exists in the United States.

This survey sheds light on the current state of SHIPPs.

## Methods

Based on the informal inquiry published by Stevens et al.,^1^ authors MS and JM created a survey together with the SHEA Research Network (SRN) team using a survey generator (Alchemer, Louisville, CO). A beta version of the questions was tested among peers and the survey further refined. The final fifty-four question survey was sent out to SRN participants in August 2023, with a reminder email sent the following month. Raw data were compiled and analyzed using Microsoft Excel.

## Results

Thirty-eight United States-based institutions responded (38/93, 41%), of which 23 had SHIPPs. These SHIPPS reported a median of 5.5 acute care hospitals (range, 1–33); 15 SHIPPS reported a median of 2 critical access hospitals (range, 1–8); 4 SHIPPs reported 1–3 long-term acute care hospitals (LTACHs), and 4 SHIPPS reported a median of 1.5 nursing homes. All except 3 (87%) included an academic center. Approximately half (11/23) operate in multiple states. Four programs have been in place >20 years, four <2 years, and the remainder a median of 7 years (range, 2–12). The complete survey results are provided as supplementary material.

### Personnel

Physician directors, in addition to IP responsibilities, also have clinical (19/23, 83%), teaching (17/23, 74%), research (14/23, 61%), antimicrobial stewardship (7/23, 30%), quality (7/23, 30%), and/or patient safety (4/23, 17%) roles. Fifteen (65%) report having a written job description. Please see Table 1 for other key medical director characteristics. The responses were noteworthy for the 17 different titles in use for the physician system IP leads.

**Table 1.** Physician director characteristics

Characteristic	***N***	Mean	Median	Distribution
Annual salary (US Dollars)	15	[Don’t have as data was in ranges]	200–249 K	100–149 K = 1 150–199 K = 1 200–249 K = 6 250–299 K = 1 ≥300 K = 6
Number of Weeks of Infectious Diseases Consult Service	15	Mean = 8.93	Median = 6	*Range* = *2–28*
Number of Half Days in Clinic per Week	13	Mean = 0.94	Median = 1	*Range* = *0–2*
How Often Medical Director Visits Sites (in Months)	21		Median = Quarterly	Do not routinely visit = 5 More than monthly = 2 Monthly = 2 Quarterly = 7 Every 6 months = 3 Yearly = 2

Eighteen (78%) report having an infection preventionist in a system IP director role; only 7/23 (30%) have a dedicated system IP team that operates independent of individual hospitals. Fourteen (61%) report administrative support, 10/23 (43%) have a data manager/analyst in their team, and 4/23 (17%) include information technology expert or programmer support.

### Resources

14/23 (61%) report having done a formal system-wide IP needs assessment. Access to system-level resources was variable (see Table 2). While a COVID-19 specific dashboard was in place for 74% of the responding organizations, the same was not true for respiratory virus dashboards, which were available to only 57% of programs.

**Table 2.** System-level infection prevention resource access

Resource	Frequency (N = 23)
Automation in HAI surveillance	61% (14)
Centralized surveillance	39% (9)
“System IP policies” that are hierarchically above individual site policies	52% (12)
System-level process metrics dashboards	43% (10)

### Challenges

The biggest challenges identified from free text survey comments include gaps in 1) clear governing structure, 2) communication across the system, 3) consistent staffing with dedicated, empowered IP experts, and 4) data management support.

## Discussion

To our knowledge, this is the first comprehensive formal survey to explore the characteristics of modern-day U.S. SHIPPs, including the structure and responsibilities of physician leaders. In our sample of hospital networks, we found heterogeneity in the structure, staffing, and resources for system IP with significant opportunities for improvement.

Barnes and colleagues surveyed a group of corporate and system-level directors of IP in 2016. Of the 32 respondents in their study, only 37% noted having physician support in their programs (vs over 85% in our study), and the training background and roles of these physicians were not described.^2^

A particular focus of our survey was in describing the job expectations, support, and remuneration of the physician epidemiologists leading or co-leading these programs. We feel that these data will be of great value to physician epidemiologists considering taking on system healthcare IP program leadership roles, particularly in the context of suboptimal compensation of ID physicians in general,^3^ and more specifically, in view of ID physicians taking on organizational roles for which they are at times not compensated at all.

Sturm and colleagues recently described their IP system program at Ascension, which operates nearly 140 hospitals in 19 U.S. states. Their leadership structure includes a system healthcare epidemiologist as well as a separate system IP leader. They note the value of a system approach to IP includes standardization, capacity building, and education.^4^ Our survey results indicate great variability across SHIPPs in terms of personnel, data infrastructure, and frontline program support. Interestingly, certain aspects suggest that large networks increase efficiency; healthcare-associated infection surveillance, for example, has been centralized in 39% of the SHIPPs represented here. Centralized surveillance represents an opportunity unique to health systems and allows for surveillance standardization and program efficiency.^5^

Beyond the need to define optimal SHIPP structure and resources, best practices for the integration of SHIPPs with other potentially system-facing programs (such as occupational health, patient safety, quality, antimicrobial, and diagnostic stewardship) have yet to be defined.

One limitation of our study is that only institutions within the SRN were included, thus limiting the generalizability of our findings. The survey was therefore (unintentionally) geared toward physician IP experts but could easily be adapted to other roles in IP. The salary data that are presented reflect total compensation and not compensation specifically related to IP activities.

In this era of healthcare consolidation, our findings highlight the urgent need to clearly define the optimal structure and resources needed for SHIPPs. Significant opportunities exist to extend the knowledge and experience of trained IP personnel across healthcare systems, but capitalizing on these opportunities will require a better understanding of the roles, training needs, and support for these leaders. Next steps to further our understanding and support of SHIPPs may include a larger survey that expands to hospital networks outside the SRN and the drafting of a white paper for system healthcare IP.

## Supporting information

Stevens et al. supplementary material Stevens et al. supplementary material

## Supplementary material

To view supplementary material for this article, please visit https://doi.org/10.1017/ice.2024.170
